# 17 years’ experience of surgical management of thoracic outlet syndrome at a district general hospital

**DOI:** 10.1308/rcsann.2023.0002

**Published:** 2023-01-13

**Authors:** BJMc Farquharson, J Collis, S Jaskani, H Bergman, B Andrews

**Affiliations:** ^1^ Medway NHS Foundation Trust, UK; ^2^ Cambridge University Hospitals NHS Foundation Trust, UK

**Keywords:** Thoracic outlet syndrome, Treatment outcome, First rib resection, District general hospital

## Abstract

**Introduction:**

Thoracic outlet syndrome (TOS) is caused by compression of the neurovascular structures passing through the thoracic inlet. It is categorised into three subtypes: neurogenic TOS (NTOS), venous TOS (VTOS) and arterial TOS (ATOS). This study evaluates the outcomes of patients who underwent first rib resection (FRR) for TOS during a period of 17 years at a single district general hospital.

**Methods:**

Retrospective review of patient notes of individuals treated with FRR from August 2004 to August 2021.

**Results:**

A total of 62 FRRs were performed on 51 individual patients. Indications for FRR included 42 NTOS (68%), 6 VTOS (10%) and 14 ATOS (23%). Thirty-four patients (64%) were female and the mean age at time of surgery was 39 years (range 27 to 64 years). Eleven patients (21%) underwent bilateral FRR and seven cases of cervical ribs were observed. The mean time from initial symptoms to diagnosis was 18 months (range 2 to 60 months). Overall, outcomes after surgery were positive across all subtypes of TOS. Based on Derkash’s classification, 52 patients (84%) reported excellent/good, 8 (13%) reported fair and 2 (3%) reported poor resolution of symptoms at 6 month follow-up. Complications included four (9%) pneumothorax, two (4%) wound infections, two (4%) haematoma, one (2%) haemothorax, three (5%) phrenic nerve complications and one (2%) brachial neuropraxia.

**Conclusions:**

FRR for TOS can be performed safely and effectively in a district general hospital environment with excellent patient clinical outcomes.

## Introduction

**Figure 1:**
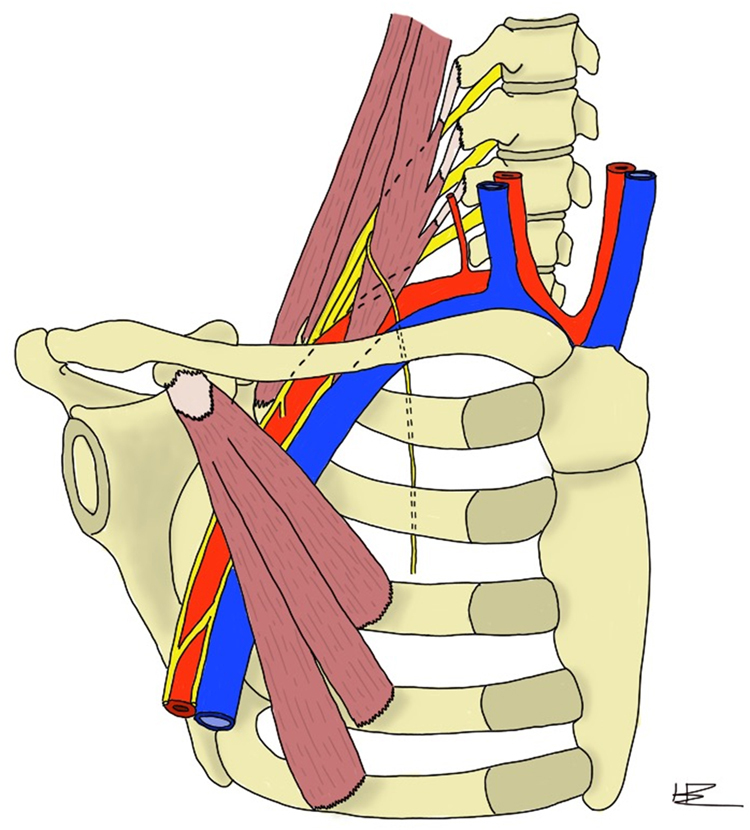
Anatomical structures of thoracic outlet

Thoracic outlet syndrome (TOS) is caused by compression of the neurovascular structures passing through the thoracic inlet (Figure 1). It is categorised into three subtypes: neurogenic TOS (NTOS), venous TOS (VTOS) and arterial TOS (ATOS). These subtypes occur secondary to compression of brachial plexus, subclavian vein or subclavian artery. Thoracic outlet compression has multiple aetiologies, it may result from anomalous ribs, congenital cervical fibro-cartilaginous bands, muscular anomalies or injury.

The most common subtype is NTOS, which accounts for approximately 95% of cases; VTOS accounts for 3% of cases and ATOS accounts for 1% of cases.^1^ Symptoms of brachial plexus compression include pain, weakness, numbness and paraesthesia of the upper limb. Venous compression may result in arm swelling and deep vein thrombosis. Arterial compression may cause upper limb claudication symptoms, embolic events and acute limb ischaemia. Symptoms may be aggravated by activities that involve prolonged use of arms or hands and elevation of upper extremity.^2^

Initial management of NTOS is physiotherapy and some centres offer adjunctive medical therapy with local anaesthetic, steroids and botulinum toxin.^3^ Patients who have failed conservative management are offered thoracic outlet decompression surgery. VTOS and ATOS warrant expedited surgical management owing to the high incidence of secondary complications. The standard for surgical care of TOS patients is first rib resection (FRR).^4^ Surgical approaches include supraclavicular, infraclavicular and transaxillary, with the choice of procedure determined by the subtype of TOS and surgeon preference. Recently, thoracoscopic (assisted) and robotic approaches have been described in the literature.^5^

This retrospective observational study aims to evaluate the outcomes of surgical management of thoracic outlet syndrome at a single district general hospital (DGH) over a period of 17 years.

## Methods

### Local protocol

In our institution, diagnosis of NTOS was determined via clinical evaluation, nerve conduction studies and magnetic resonance imaging (MRI) cervical spine. MRI cervical spine is performed routinely in this patient cohort. Nerve conduction studies are performed in cases of diagnostic uncertainty to rule out common differential diagnoses such as carpal tunnel syndrome. Patients with NTOS are offered physiotherapy first line, and cases refractory to conservative management proceed to surgery. NTOS patients are reassessed after completion of physiotherapy in a consultant surgeon outpatient clinic before planned surgical intervention. Adjunctive medical therapy is not offered at our institution. Diagnosis of VTOS is determined via a combination of clinical evaluation, duplex ultrasonography and angiographic imaging studies. Cases of VTOS are treated with anticoagulation and catheter-directed thrombolysis followed by expedited surgical management. Confirmed cases of ATOS proceed to surgery.

Supraclavicular approach FRR and scalenectomy was performed on patients with NTOS and ATOS in this study. Infraclavicular approach FRR and scalenectomy was performed on all cases of VTOS. Adjunctive cervical rib resection was completed when indicated. Routine use of the minimal traction technique, bipolar cautery and nerve stimulation equipment was employed intraoperatively to reduce risk of iatrogenic nerve injury. Bilateral FRR is performed as two-stage procedure with stages a minimum of 12 months apart and the more symptomatic side operated on in the first instance. All patients completed postoperative outpatient follow-up of at least 6 months.

### Data collection

Data collection was completed via retrospective review of patient notes at a single DGH. All patients who underwent FRR for TOS from August 2004 to August 2021 were included in the study. Information was gained from the local clinical coding team who identified patients through the general surgery elective list database. Patient notes were obtained via the health records team within the clinical audit department. Patients included in this study were operated on by one consultant surgeon within the general surgical department at the DGH.

A proforma was created to streamline data collection. Demographic and clinical characteristics included: age, sex, laterality, subtype, aetiology, length of time from initial symptoms to diagnosis, postoperative complications, length of stay and clinical outcome. Clinical outcome was determined via Derkash’s classification validated patient-reported outcome measure.^4,6^ Derkash’s classification is a post-procedural assessment of severity of symptoms of TOS. It offers an appraisal of the ability to return to activities of daily living and professional commitments (Table 1). For statistical analysis, IBM SPSS Statistics for Windows, version 23.0. (IBM Corp., Armonk, NY, USA) was used. Informed consent was obtained from each patient for inclusion in this study.

**Table 1:** Derkash’s classification

***Classification***	**Description**
*Excellent*	No pain, easy return to preoperative professional and leisure daily activities.
*Good*	Intermittent pain well tolerated, possible return to preoperative professional and leisure daily activities.
*Fair*	Intermittent pain with bad tolerance, difficult return to preoperative professional and leisure daily activities.
*Poor*	Symptoms not improved or aggravated.

## Results

A total of 62 FRRs were performed on 51 individual patients at a single DGH between August 2004 and August 2021. Indications for FRR included 42 NTOS (68%), 6 VTOS (10%) and 14 ATOS (23%). Thirty-four patients (64%) were female and the mean age at the time of surgery was 39 years (range 27 to 64 years). The majority of this patient cohort were American Society of Anesthesia (ASA) grade 1 (79%). The remaining patients were ASA grade 2 (16%) and ASA grade 3 (6%), with no ASA grade 4 patients undergoing FRR for TOS at our centre. There were similar numbers of right (*n* = 33) and left (*n* = 29) TOS, and there was no association between laterality and subtype of TOS (Table 2). Eleven patients (21%) underwent bilateral FRR. Seven cases of cervical ribs were observed, which were associated with four cases of NTOS and three cases of ATOS. The mean time from initial symptoms to diagnosis was 18 months (range 2 to 60 months). Forty patients (95%) with NTOS initially completed physiotherapy before proceeding to definitive surgical management. Two NTOS patients were not offered physiotherapy because they had previously completed a course of physiotherapy via community services prior to referral.

**Table 2:** Subgroup analysis of demographic data

	**Neurogenic (*N *= 42)**	**Arterial (*N* = 14)**	**Venous (*N *= 6)**
	*n* (%)	*n* (%)	*n* (%)
*Male*	14 (33.3)	9 (64.3)	3 (50.0)
*Female*	28 (66.7)	5 (35.7)	3 (50.0)
*ASA 1*	35 (83.3)	9 (64.3)	5 (83.3)
*ASA 2*	6 (14.3)	3 (21.4)	1 (16.7)
*ASA 3*	1 (2.4)	2 (14.3)	0 (0)
*ASA 4*	0 (0)	0 (0)	0 (0.0)
*Left*	18 (42.9)	8 (57.1)	3 (50.0)
*Right*	24 (57.1)	6 (14.3)	3 (50.0)
*Cervical rib(s)*	4 (9.5)	3 (21.4)	0 (0)

Postoperatively, the mean length of stay for each patient was 1.6 days (range 1 to 5 days). Overall, outcomes after surgery were positive across all subtypes of TOS. Based on the Derkash’s classification, 52 (84%) reported excellent/good, 8 (13%) reported fair and 2 (3%) reported poor resolution of symptoms at 6-month follow-up. The most common complication was pneumothorax with four cases (6%). Other complications included two (4%) wound infections, two (4%) haematomas, one (2%) haemothorax, three (5%) phrenic nerve complications and one (2%) brachial neuropraxia. Two patients with phrenic nerve injury symptoms self-resolved without intervention. One patient with phrenic nerve injury underwent video-assisted thoracoscopic surgery diaphragmatic plication procedure with resultant resolution of symptoms. Subgroup analysis was performed to assess differences in outcomes between subtypes of TOS (Tables 3 and 4). There was no significant difference between clinical outcome or complication rate between subtypes of TOS (*p *> 0.05).

**Table 3:** Subgroup analysis of postoperative outcomes

	**Neurogenic (*N *= 42)**	**Arterial (*N *= 14)**	**Venous (*N *= 6)**
***Derkash*** c***lassification***	*n* (%)	*n* (%)	*n* (%)
*Excellent/Good*	33 (78.6)	13 (92.9)	6 (100)
*Fair*	7 (16.7)	1 (7.1)	0 (0)
*Poor*	2 (4.8)	0 (0)	0 (0)

**Table 4:** Subgroup analysis of postoperative complications

	**Neurogenic (*N *= 42)**	**Arterial (*N *= 14)**	**Venous (*N *= 6)**
	***n* (%)**	***n* (%)**	***n* (%)**
*Pneumothorax*	3 (7.1)	0 (0)	1 (16.7)
*Wound infection*	1 (2.4)	1 (7.1)	0 (0)
*Haematoma*	2 (4.8)	0 (0)	0 (0)
*Haemothorax*	1 (2.4)	0 (0)	0 (0)
*Brachial neuropraxia*	1 (2.4)	0 (0)	0 (0)
*Phrenic nerve complication*	2 (4.8)	1 (7.1)	0 (0)

## Discussion

This study appraises clinical outcomes of the surgical management of NTOS, VTOS and ATOS at a single centre over a 17-year period. We present positive outcomes of FRR across all three subtypes of TOS. This series is the first of its kind to assess outcomes of patients undergoing FRR in a DGH environment in the United Kingdom. This study indicates that surgery for TOS in an adult population can be performed safely and effectively in this setting.

One-third of patients who present with NTOS fail conservative management and proceed to surgery.^7^ The literature suggests that surgical management of NTOS leads to a significant improvement in patient-reported quality-of-life metrics and that this is sustained on long-term follow-up.^8,9^ This series revealed 78.6% of patients who underwent FRR for NTOS resulted in Derkash’s classification of good or excellent outcomes. Fair and poor outcomes in our patient cohort were associated with increasing age and ASA. Risk factors for poor outcomes after surgery for NTOS include smoking, age, chronic opiate use and comorbidities.^9^ Davoli *et al* state that patient selection is the key determinant of success in the surgical management of NTOS.^10^ In our experience, careful investigative work-up is necessary to prevent misdiagnosis in this patient group.

VTOS presents with upper extremity venous thrombosis, historically known as Paget–Schroetter syndrome. Initial treatment with catheter-directed thrombolysis has been shown to be almost 100% successful if performed within 2 weeks of diagnosis.^11^ Recent studies have recommended early FRR post thrombolysis owing to a risk of venous reocclusion.^12–14^ Conservative management with long-term anticoagulation is an alternative treatment option in this cohort of patients. Follow-up imaging is essential to assess the patency of the venous system and to assess the requirement for further intervention with both conservative and surgical management. Sheth and Belzberg purport an association of VTOS with repetitive strain injury and thus an association with occupation and hand dominance.^15^ This is reflected in our data set with VTOS associated with occupations such as labourers and athletes.

This series presents a high proportion of ATOS in comparison with the accepted demographic analysis of TOS.^1^ This is likely reflective of difficulty in the diagnosis of NTOS and the relative ease of diagnosis of VTOS owing to its clinical presentation and access to diagnostic imaging. Prompt surgical management of ATOS offers excellent clinical outcomes.^4^ Conservative management has no place in cases of arterial compression because of high likelihood of secondary complications and risk of acute limb ischaemia. Of note, NTOS and ATOS may coexist. Comprehensive clinical assessment is essential for accurate diagnosis and management.

**Figure 2:**
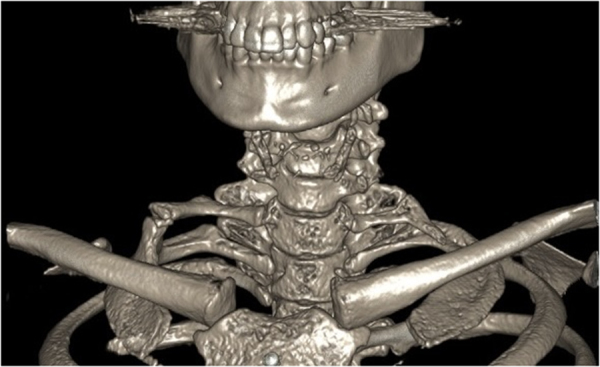
Three-dimensional reconstruction of cervical ribs

The literature suggests that 10% of patients with cervical ribs develop TOS (Figure 2). Cervical ribs are more likely to be associated with NTOS and ATOS, and this is reflected in the results of this study (Table 2). Cervical ribs are associated with more frequent secondary complications of ATOS including aneurysm, stenosis or occlusion.^3^ This patient cohort requires aggressive surgical management with resection of the cervical rib and first rib to successfully treat thoracic compression.

A supraclavicular approach is recognised as the most appropriate technique for arterial decompression surgery because it allows better exposure and control of the subclavian artery.^16^ All patients in our centre underwent this approach for FRR and scalenectomy in all cases of ATOS and NTOS. In our experience, an infraclavicular approach provides optimal access to the subclavian vein and thus is utilised for all patients presenting with VTOS. This method is supported by a recent systematic review revealing an overall secondary venous patency rate of 98.5% with the use of an infraclavicular approach for VTOS.^17^ A transaxillary approach is preferred in some centres owing to its superior cosmetic results and it does not require division of musculature.^18^ The surgical approach should be determined via patient anatomy and surgeon preference. All techniques for surgical decompression of thoracic outlet have positive results in expert hands.

**Figure 3:**
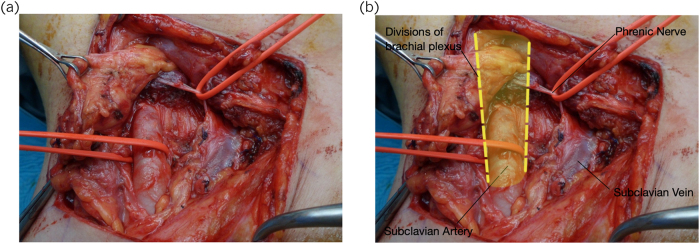
(a) Right supraclavicular exposure. (b) Right supraclavicular exposure (labelled)

Complication rates described in the literature vary between 5% and 40%, with pneumothorax, nerve injury and wound infection reported as the most commonly occurring complications.^4^ Supra- and infraclavicular approaches provide clear exposure to neurovascular structures of the thoracic inlet and literature suggests that this results in a reduced incidence of brachial neuropraxia.^19^ This is supported by this series with only one episode recorded in our patient cohort. This study revealed three cases of phrenic nerve complications with use of the supraclavicular approach. In this technique, the phrenic nerve is at risk when the anterior scalene muscle is divided to gain access to thoracic outlet neurovascular structures (Figure 3). Injury to the phrenic nerve may result in paralysis of the ipsilateral diaphragm. Two cases of phrenic nerve injury were self-resolving. A single case required referral to cardiothoracic centre for diaphragmatic plication with complete resolution of symptoms. Urgent cardiothoracic opinion should be sought in any case of phrenic nerve complication.

A minimal traction technique on spinal nerves, phrenic nerve and brachial plexus trunk was employed intraoperatively to attempt to avoid iatrogenic nerve injury. In all cases in this series, the phrenic nerve was identified using a nerve stimulator and slung prior to division of the scalenus anterior muscle (Figure 3). In no case was the nerve divided and despite precautions such as the use of bipolar diathermy to divide the scalenus muscle we noted three nerve palsies. We postulated that these complications may arise from axonotmesis or neurotmesis due to tissue handling or loss of microvascular blood supply in dissection. Furthermore, aberrant phrenic nerve anatomy not picked up intraoperatively may also have contributed to this result. Phrenic nerve injury is an uncommonly reported complication in the literature and patients should be counselled appropriately on the potential of injury before proceeding to operative management of TOS.

This study demonstrates the diagnostic challenge associated with TOS by the prolonged time from initial symptoms to diagnosis, with patients waiting on average 18 months before receiving a definitive diagnosis. The diagnosis of NTOS is often one of exclusion with investigations frequently more useful to rule out differential diagnoses than offering clear answers to the clinical question. VTOS frequently presents in the acute setting and is more readily diagnosable on imaging. TOS presents a challenge to primary care with no clear national pathway or guidance for referral to secondary care. Patient referrals in our hospital are often received from subspecialities including neurologists, orthopaedics and pain specialists. High-volume centres in the USA have clear pathways in place for assessment with resultant strongly positive outcomes.^3^ The UK has no such system in place and undoubtedly would benefit from development of centres of excellence with clear referral pathways. This study has revealed TOS can be managed safely and effectively in a DGH environment. Thus, providing evidence centres of excellence may be reproducible outside of tertiary centres.

### Study limitations

This study has limitations because it is a single-centre, retrospective, observational clinical notes review of practice. Despite best efforts in uniformity of data collection, it may be subject to bias. Nonetheless, the study maintains external validity in that data are taken from a high-volume general and vascular surgical department broadly comparable with most centres across the UK. Furthermore, this series provides a novel assessment of surgical practice for TOS within the UK with no comparable study published to date in the wider literature.

## Conclusion

FRR for TOS can be performed safely and effectively in a DGH environment with excellent patient clinical outcomes. This series presents the difficulty of diagnosis of TOS with patients often waiting prolonged periods before receiving definitive treatment.
